# Reply to Sun: Behavior affects epidemic outcomes

**DOI:** 10.1073/pnas.2618282123

**Published:** 2026-07-13

**Authors:** Simon K. Schnyder, Mark P. Lynch, John J. Molina, Ryoichi Yamamoto, Matthew S. Turner

**Affiliations:** ^a^ Institute of Industrial Science, The University of Tokyo, Tokyo 153-8505, Japan; ^b^ Mathematics for Real-World Systems Centre for Doctoral Training, Department of Mathematics, University of Warwick, Coventry CV4 7AL, United Kingdom; ^c^ Department of Chemical Engineering, Kyoto University, Kyoto 615-8510, Japan; ^d^ Department of Physics, University of Warwick, Coventry CV4 7AL, United Kingdom; ^e^Institute for Global Pandemic Planning, Coventry CV4 7AL, United Kingdom

We recently predicted how partially altruistic individuals would social distance during an epidemic of an infectious disease (1).

We encoded the preferences of altruistic individuals in an objective function, containing costs to the individuals and others. We found that well-informed populations possessing an altruism above a critical threshold can spontaneously choose from two equilibria, one of which results in far lower disease prevalence. Here, we address criticism of this work by Sun (2).

Sun observes that many other behaviors affect disease transmission, e.g., mask wearing. This is a fair point and we could have listed more examples. However, making such distinctions affects neither model nor conclusions, as Sun acknowledges: any costly transmission-reducing behavior enters the analysis similarly. Our framing then also applies to animals, as they do not wear masks but could plausibly evolve to social distance.

Sun’s argument is that different behavioral equilibria need not produce different epidemiological outcomes. However, we set out to study how well-informed rational individuals would act if they knew the effects and costs of their actions. We could have assumed that adjusting their behavior is costly but ineffective or that individuals are unaware of the true costs of their actions; in these cases, the population might choose not to adjust its behavior.

Contrary to Sun’s suggestion, we neither assumed that increased altruism would automatically yield “improved infectious dynamics” (2) nor that it would even change a person’s behavior. Even at high altruism, individuals need not social distance if the rest of the population does not. The critical altruism threshold is not encoded directly in the objective function but emerges at the point where the outbreak can be spontaneously contained by the population.

Sun questions our modeling assumptions, in particular the nonlinear social distancing cost. We did not assume a linear form because marginal costs tend to increase as behavior becomes more extreme: otherwise, reducing one’s social activity from 100 to 50% would carry the same cost as reducing it again down to 0%, which seems unrealistic.

Our modeling choices are robust to variation: the Indefinite Suppression equilibrium qualitatively survives large changes to the cost of infection, the value of *R*_0_, the epidemic duration, and a moderate amount of asymptomatic cases. We believe it would survive changing the cost’s functional form to linear (albeit generating a bang–bang type solution) or other nonlinear forms: the main requirement would seem to be that reducing social contacts to a level ~1/*R*_0_ remain accessible.

Sun’s modeling proposal, “a focal agent considers herself to be worth n other individuals if and only if her probability of remaining susceptible balances their risk of infection,” is not precise enough for us to be able to analyze it.

Despite Sun’s claims of us studying a few “highly complex models,” we argue that ours is the simplest model in which one can sensibly study the effect of altruism on self-organized behavior in an epidemic.

## Data Availability

There are no data underlying this work.
